# Virtual realities, real recoveries: exploring the efficacy of 3MDR therapy for treatment-resistant PTSD

**DOI:** 10.3389/fpsyg.2024.1291961

**Published:** 2024-05-09

**Authors:** Åsmund Gausemel, Petra Filkuková

**Affiliations:** ^1^Department of Psychology, Inland Norway University of Applied Sciences, Lillehammer, Norway; ^2^Department of High Performance Computing, Simula Research Laboratory, Oslo, Norway

**Keywords:** multimodular motion-assisted memory desensitization and reconsolidation (3MDR), treatment-resistant posttraumatic stress disorder (TR PTSD), exposure therapy, virtual reality, moral injury

## Abstract

Exposure-based therapies have shown promise in treating post-traumatic stress disorder (PTSD), but challenges exist in maintaining patient engagement and finding appropriate stimuli for graded exposure. Virtual reality (VR) technology has been used to enhance exposure therapy, but current software lacks customization and some patients remain treatment-resistant. A novel approach called multimodular motion-assisted memory desensitization and reconsolidation (3MDR) has the potential to solve some of the current limitations of VR-assisted exposure therapy. This study examines the efficacy of 3MDR treatment for individuals with treatment-resistant PTSD through a systematic review of relevant literature and clinical studies. Preliminary findings indicate promise for 3MDR in reducing PTSD symptoms, including emotional regulation and moral injury. However, further research with larger samples and controlled studies is needed to understand underlying mechanisms and validate these results. Moreover, this study highlights the importance of health-economic evaluations to assess costs and resource utilization associated with implementing 3MDR treatment in clinical services.

## 1 Introduction

A typical response to traumatic events involves several predictable stress reactions: re-experiencing the event (flashbacks), avoidance of similar stimuli, and hyperarousal, which typically subsides within a few days or weeks after the trauma (World Health Organization, 1992, pp. 147–148). However, there is a significant minority of individuals who experience that these reactions persist; therefore, these responses remain as symptoms of post-traumatic stress disorder (PTSD).

Historically, war has been a leading cause of PTSD (Friedman et al., 2007). Soldiers have experienced the traumatic effects of warfare throughout the various conflicts in history. PTSD-like symptoms have been labeled under several different names, but PTSD was not officially recognized as a separate diagnosis until the 1980s (Friedman et al., 2007). For instance, during the American Civil War, traumatized soldiers were said to have “soldier’s heart” or “Da Costa’s syndrome” (Friedman et al., 2007, p. 20). As more powerful explosives were developed during World War I, the term “shell shock” was used to describe what was believed to be disruptions in neural networks. This term evolved to “combat fatigue” during World War II, and after the Vietnam War, the condition was recognized to affect both civilians and soldiers who had experienced trauma as a consequence of warfare (Friedman et al., 2007, p. 20). There was a high prevalence of PTSD symptoms among those with war experience, leading to the official recognition of PTSD as a separate diagnosis in the Diagnostic and Statistical Manual of Mental Disorders (DSM-III) in 1980 (Friedman et al., 2007).

### 1.1 Exposure based methods

Behaviorist psychologists viewed mental disorders as continuous attempts to avoid confrontation with fear-inducing stimuli (Mowrer, 1960). Despite the theoretical differences between behaviorism and other approaches to psychotherapy, a common principle for treating PTSD symptoms has been developed across psychotherapeutic schools: the principle of exposure. If PTSD symptoms persist due to avoidance behavior, where patients fail to recognize and/or process discomforting information, psychotherapy can be interpreted as a setting in which confrontation with such information is enabled to facilitate change. Psychodynamically oriented therapists, for example, expose their patients to information about unconscious conflicts, painful memories, and unacceptable desires through the interpretation of their behavior in therapy, dreams, or free associations (Friedman et al., 2007, pp. 37–40). More recent forms of psychotherapy specifically developed for anxiety disorders involve a more direct focus on exposure.

There are numerous psychological treatment methods for PTSD, and a recent systematic review and meta-analysis by Lewis et al. (2020) suggests that trauma-focused cognitive-behavioral therapy (TF-CBT), cognitive processing therapy (CPT), prolonged exposure therapy (PE), and eye movement desensitization and reprocessing therapy (EMDR) demonstrate efficacy (Lewis et al., 2020). What these methods have in common is a form of exposure-based methodology. Exposure therapy can be conducted in various ways, but the essence of exposure-based treatment methods involves gradual exposure to anxiety- or fear-inducing stimuli while working to reduce the negative reactions that may arise so that these stimuli are no longer associated with fear (Zoellner et al., 2011, pp. 2–3). For instance, an individual with arachnophobia may be gradually exposed to pictures of spiders, then real spiders at a distance, and eventually handle a spider in a controlled manner.

A challenge associated with exposure-based methods appears to be that many individuals struggle to envision traumatic events during imaginal exposure (Najavits, 2015). Avoiding thoughts of what one fears is easier during imaginal exposure and has proven to be problematic. Furthermore, stimuli used for gradual exposure can sometimes become overwhelming and result in drop-out. Finding real-life stimuli suitable for systematic and gradual exposure, especially for military veterans, can be challenging (Najavits, 2015) Several studies have reported dropout rates and non-response rates as high as 50% for EMDR, PE, and cognitive processing therapy (Schottenbauer et al., 2008; Najavits, 2015). This is unfortunate, as emotional activation and engagement appear to be crucial for treatment outcomes in populations with PTSD (Foa et al., 2006).

### 1.2 Virtual exposure therapy

Virtual reality exposure therapy (VRET) has emerged as a promising alternative to conventional exposure-based methods, offering opportunities to simplify and enhance exposure. VRET provides a human-computer interaction system where users are no longer passive observers of images on a screen but active participants within a computer-generated three-dimensional virtual world (Rothbaum et al., 2010, p. 127). The most common approach to creating a virtual environment involves equipping the user with a head-mounted display comprising separate screens for each eye, screen optics, a head-tracking sensor, and stereo headphones (Rothbaum et al., 2010, p. 127). VRET has the potential to address the difficulties many individuals face in imagining traumatic memories and therefore increase therapeutic engagement. It also addresses the problem of identifying appropriate stimuli for graded exposure. When using VRET, the only limiting factor is the software, which can be tailored to create virtual environments specific to individual participants.

Recent meta-analyses and systematic literature reviews suggest that VRET exhibits comparable efficacy to traditional imaginal exposure therapies for PTSD treatment (Deng et al., 2019; Kothgassner et al., 2019; Heo and Park, 2022). However, there is no evidence to suggest that VRET outperforms other exposure methods. Therefore, whether VRET is a better alternative becomes a matter of cost-effectiveness. Nonetheless, VRET can be considered a viable option for those who struggle with envisioning traumas (Heo and Park, 2022). Customizing software for each client is a time-consuming and potentially cost-ineffective process. Consequently, software has been developed where clinicians can add “typical” elements related to PTSD traumas, such as elements from war zones (explosions or gunfire) (Rothbaum et al., 2010). However, questions remain regarding how well these elements align with the specific trauma memories of each individual client (van Gelderen et al., 2018). The use of head-mounted displays in VRET also poses the risk of isolating participants from the external environment. Dropout rates in VRET studies remain relatively high, and there is a lack of knowledge regarding the optimal level of realism and immersion for effective treatment (Kitchiner et al., 2019). The experience of isolation in VRET may lead to feelings of inadequate support, trigger dissociation, or result in higher drop-out rates.

### 1.3 The need for a new treatment approach

Various evidence-based therapies, such as Eye Movement Desensitization and Reprocessing (EMDR), Virtual Reality Exposure Therapy (VRET), and Prolonged Exposure (PE), have demonstrated significant effect sizes in the treatment of posttraumatic stress disorder (PTSD). However, it is consistently observed that some clients do not respond to therapy, even after repeated attempts and different therapeutic approaches (Kitchiner et al., 2019; Lewis et al., 2020). This phenomenon has been increasingly referred to as treatment-resistant PTSD (TR-PTSD) (Koek et al., 2016, p. 173). The reasons why certain individuals exhibit this resistance to treatment remains poorly understood, and few studies have specifically focused on samples consisting of individuals with TR-PTSD (Koek et al., 2016).

**Figure 1:**
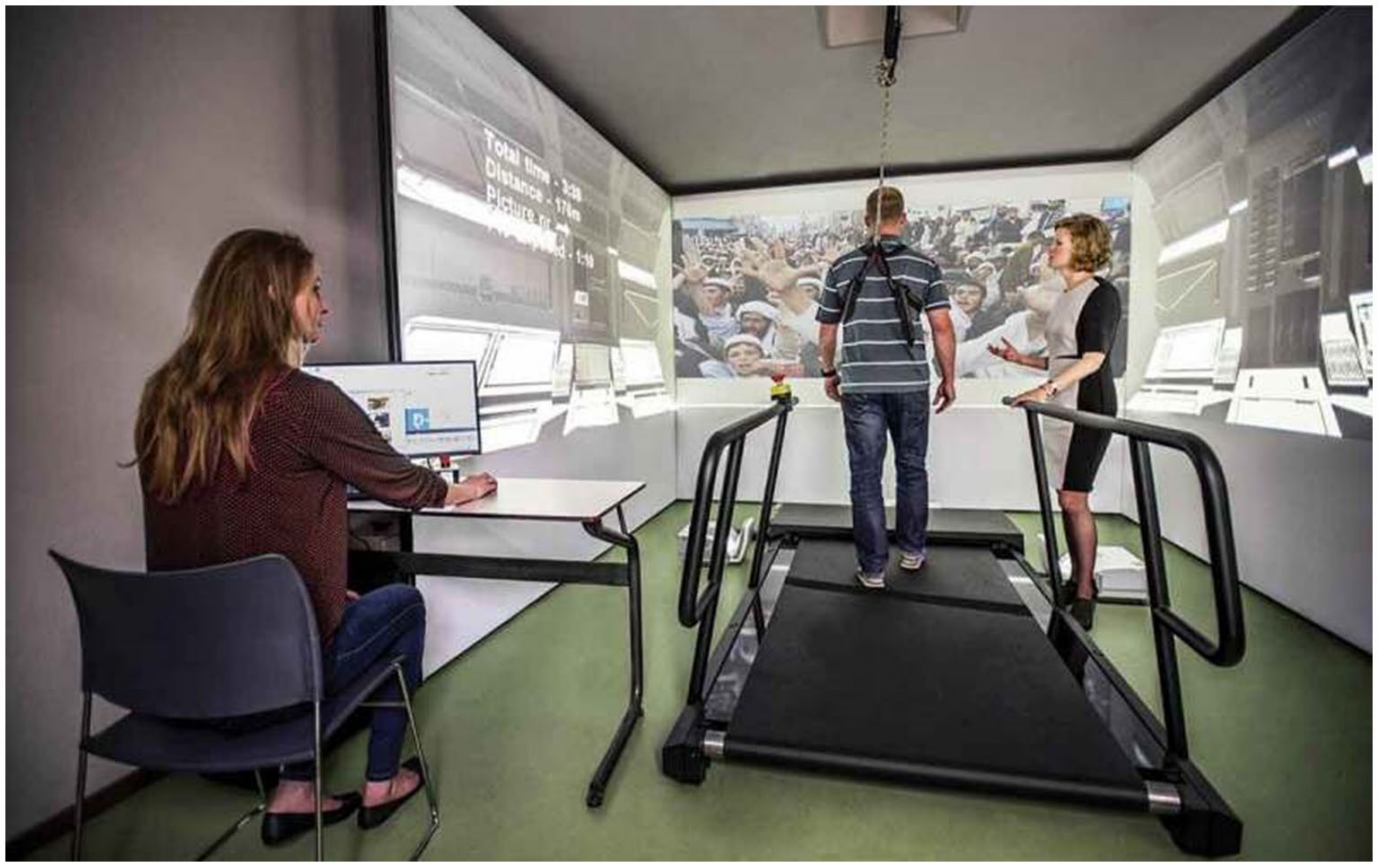
Digital illustration of the “CAVE” 3MDR setup (van Gelderen et al., 2020b, CC BY-NC 4.0).

Consequently, there is a pressing need for new treatment alternatives that can be proposed for the management of PTSD, particularly TR-PTSD. One novel exposure therapy known as Multi-Modular Motion-assisted Memory Desensitization and Reconsolidation (3MDR) may be a good candidate to solve this problem (Figure 1).

### 1.4 The 3MDR protocol

Multimodular motion-assisted memory desensitization and reconsolidation represents a novel intervention specifically developed for individuals with TR-PTSD, combining elements of VRET and EMDR, along with the integration of walking on a treadmill and interacting with personally selected images and music (van Gelderen et al., 2018, p. 6). This approach holds promise as an alternative to VRET and methods utilizing imaginal exposure, as 3MDR appears to address some of the limitations associated with VRET. In 3MDR, participants are situated within a screen room alongside a therapist, thus eliminating the sense of isolation from the external environment that might be experienced in VRET. By creating a virtual environment where the patient and therapist can visually perceive each other, attention within the therapeutic setting can be enhanced without the risk of isolating the patient from the real world. Moreover, 3MDR facilitates greater customization of the therapeutic experience for each individual, as there is no need for software modifications to project participant-selected music and images.

The 3MDR sessions consist of three distinct phases: pre-platform, platform, and post-platform (van Gelderen et al., 2018, pp. 8–9). During the pre-platform phase, the therapist and participant collaborate to select appropriate images and music to be utilized throughout the session. The objective here is to choose music and images that have relevance to the participants’ traumas and difficulties. However, the selection also includes music with a reconciliatory effect, which is intended to be used toward the end of the 3MDR protocol.

Topics related to avoidance concerning certain images are discussed with the therapist, and participants are guided on how best to contribute to the selection of suitable materials (van Gelderen et al., 2018, pp. 8–9). Following a thorough explanation of this task, participants choose images and music at home. They then bring their choices to preparatory sessions and, in collaboration with the therapist, determine which images and music are most appropriate and if any elements are missing.

The platform phase within each 3MDR session commences with a mental and physical warm-up, during which the patient walks on the treadmill in a neutral virtual environment while listening to music related to their trauma (van Gelderen et al., 2018, pp. 8–9). Subsequently, a cycle of approximately 5 min is repeated seven times, where in total seven different trauma-related images are shown to the participant. Throughout this cycle, the participant walks toward an approaching image, while the therapist, standing beside the participant, encourages them to disclose their thoughts and feelings associated with the image. Participants are prompted with questions such as “What are you feeling now?” to which they may respond, for example, “sadness,” and these responses are displayed on the screen. All associations described are captured as they are transcribed by the session operator and projected on the screen. Following the participant’s account of the traumatic event, a dual-attention task is introduced (van Gelderen et al., 2018, pp. 8–9). In this task, participants are asked to track a horizontally moving ball on the screen with their eyes while simultaneously verbalizing the numbers displayed on the ball. This is followed by participants providing a stress rating of their experienced distress. After seven cycles, a mental relaxation phase ensues, accompanied by the playing of contemporary music. As mentioned, this cycle is repeated seven times before the participant enters the final phase.

In the post-platform phase, a patient and a therapist dedicate time to reflect on the session and focus on significant or newly emerged cognitive and emotional associations, aiming to further integrate this information into the participant’s current life situation (van Gelderen et al., 2018, pp. 8–9).

### 1.5 Theoretical rationale for 3MDR and exposure

Multimodular motion-assisted memory desensitization and reconsolidation incorporates multiple elements, each of which is included based on a strong theoretical foundation. In its development, 3MDR draws inspiration and combines principles from various trauma-focused therapies such as PE, EMDR, and VRET. While these therapeutic approaches share a theoretical basis, they differ in their practical application, each making its unique contributions. The common thread among these therapeutic modalities is the intention to activate traumatic memories through exposure, followed by facilitating new learning or alteration/organization of the memory. Although the type of exposure and its execution may vary, the goal remains the same.

#### 1.5.1 Reconsolidation theory

For a long time, it was believed that memories once consolidated (stored) would be difficult to disrupt and would have a permanent and unalterable trace (Glickman, 1961).

However, subsequent research suggested that when a memory is reactivated, it becomes temporarily destabilized and malleable (McGaugh, 1966). These findings gave rise to the theory of reconsolidation. Further research revealed that memory activation is followed by a protein synthesis-dependent restabilization period, during which reconsolidation (re-storage) of the memory (and the associated learning) must occur for it to persist (Nader et al., 2000). Specifically, concerning fear memories, it has been observed that they require protein synthesis in the amygdala to form a new memory trace after reactivation for reconsolidation to take place (Nader et al., 2000). Subsequent meta-analyses examining research on reconsolidation theory provide strong support for the theory (Vermetten et al., 2014; Schroyens et al., 2023). In the context of PTSD treatment, the aim is therefore to first activate the traumatic memory and then introduce new learning or reorganization into the memory before it is restabilized (reconsolidated).

#### 1.5.2 Emotional processing theory

This relates to what is known as Emotional Processing Theory (EPT), which emerged shortly after reconsolidation theory gained increasing support (Foa and Kozak, 1986). EPT proposes that PTSD is caused by pathological fear schemas/structures that can arise if the schema of the fear event (trauma) is poorly organized or contains overly strong associative learning that generalizes to related, but not identical, stimuli (Foa et al., 2006). These fear structures are believed to be poorly integrated to the individual’s autobiographical memory as they exhibit a pathological and fragmented organization (Foa et al., 2006). Therefore, accessing these fear structures is challenging. It is suggested that one needs to emotionally activate the individual, similar to the original traumatic event, in order to modify or organize the fear structures. This theory aligns with reconsolidation theory as it requires activation of the “memory” to facilitate change but builds on the theory by adding the concepts of isolated fear schemas. However, the research in this area is somewhat ambiguous as some studies propose that change occurs when there is modification to the original fear structure, while others suggest the formation of new “healthier” fear structures (through new associative learning) that inhibits the previous fear structure (Dunsmoor et al., 2015; Borgomaneri et al., 2021). Nonetheless, in clinical practice, it is observed that emotional activation and the activation of memory associations are crucial for treatment outcomes, providing support for the reconsolidation paradigm (Rauch and Foa, 2006; Zoellner et al., 2011). As suggested by van Gelderen et al. (2018), the therapeutic aspects of 3MDR can be related to reconsolidation theory. Each element serves a purpose, with some increasing the likelihood of activating memory associations while others aim to disrupt or strengthen memory consolidation.

### 1.6 Aims

While many exposure-based therapy approaches demonstrate effectiveness, a significant proportion of patients do not benefit from these treatments, leading to drop-out and are therefore labeled as having treatment-resistant PTSD. There is a lack of research on treatment methods that are effective for this specific population. This reflects the need for new treatment alternatives or methods for individuals with treatment-resistant PTSD. The aim of this review is to examine whether 3MDR can assist individuals with treatment-resistant PTSD. Additionally, we will explore the mechanisms of action underlying 3MDR in light of the studies presented in the results section and the theories introduced in the introduction.

## 2 Method

In order to find relevant articles, we used Google Scholar and the Norwegian academic search engine Oria^1^. The initial search for 3MDR treatment did not prove to be specific enough (as MDR refers also to multidrug resistance and 3MDR is a name of an Australian radio station) and was therefore further refined to search words *3MDR effects on PTSD*; *3MDR for treatment-resistant PTSD*; *3MDR for chronic PTSD* and *3MDR qualitative study*. Additionally, the snowball method was applied, which involved reviewing the reference lists of relevant studies in order to discover additional articles. Due to 3MDR being a relatively new therapeutic approach, there is limited research on the topic, allowing for a comprehensive review of all published literature on 3MDRs effect on TR-PTSD thus far. All identified articles were recent peer-reviewed studies. Most (11 out of 13) of the articles on 3MDR align with the research question, as all published articles on 3MDR examine treatment-resistant PTSD. Conducting research on 3MDR is expensive and resource-intensive, which may have contributed to the limited number of studies. The field of research is also relatively new, resulting in the exclusion of only two articles. These were excluded because their measurements were deemed not sufficiently relevant to the research question. These articles were by Jones et al. (2022b), which examined the impact of 3MDR on the therapists administering the treatment (findings of this study are mentioned in our discussion) and van Deursen et al. (2021), which investigated heart rate and respiratory rate during 3MDR therapy.

Our review aims to comprehensively cover the existing literature on 3MDR for treatment resistant PTSD. Given the limited number of studies available, totaling 11 articles on the subject, all relevant literature was included in our review. This deliberate approach ensures that our review is exhaustive, leaving no pertinent studies excluded. The inclusive nature of our methodology eliminates the possibility of bias in study selection, as every piece of literature within the defined scope has been considered.

## 3 Results

A total of 11 research articles were included. The majority of the included studies have examined the effects of 3MDR on TR-PTSD through quantitative measurements. However, several studies have employed mixed methods, combining both quantitative and qualitative measurements. Furthermore, it was considered relevant to include case studies and qualitative studies that provide insights into individual experiences of 3MDR, as well as perceived effects or mechanisms of action. A few studies included in the analysis also focused on how PTSD-related concepts (such as moral injury and emotion regulation) correlate with reductions in PTSD symptoms after 3MDR. We categorized the articles into three main themes: the effect of 3MDR treatment, individual experiences, and potential underlying mechanisms (Table 1).

**Table 1:** Studies on 3MDR.

Authors	Aim	Participants	Sample size	Design	Results
Jetly et al. (2017)	Examine the potential of 3MDR as a treatment option for TR-PTSD.	Canadian soldiers with TR-PTSD	*N* = 8	Pilot study, pretest-posttest	Improvement in PTSD symptoms, social functioning, suppression of thoughts, avoidance behavior, and anxiety symptoms.
Bisson et al. (2020)	Investigate the effect of 3MDR on TR-PTSD.	British soldiers with TR-PTSD	*N* = 42	Randomized waitlist-controlled study	Significant improvement in PTSD symptoms with a medium to large effect size (*d* = 0.65) that persisted 26 weeks later.
Van Gelderen et al. (2020a)	Investigate the effect of 3MDR on TR-PTSD.	Dutch soldiers with TR-PTSD	*N* = 43	Randomized study with active control group	Significant reductions in PTSD symptoms with a large effect size (*d* = 0.83) that persisted at 16 weeks follow-up.
Jones et al. (2022a)	Investigate the effect of 3MDR on TR-PTSD.	Canadian soldiers with TR-PTSD	*N* = 11	Pilot data from a larger study	Significant improvement in PTSD symptoms, moral injury, depression, anxiety, and emotion regulation. Improvement persisted 6 months later.
Van Gelderen et al. (2018)	Examine individual experiences and effects of 3MDR in individuals with TR-PTSD.	Dutch soldiers	*N* = 3	Case study	Luke showed a 25-point reduction in PTSD symptoms and no longer met the criteria for PTSD. Nick showed an eight-point increase in PTSD symptoms. Peter showed a 30-point reduction and no longer met the criteria for PTSD.
Nijdam and Vermetten (2018)	Investigate individual experiences with 3MDR.	Dutch soldier with TR-PTSD	*N* = 1	Case study	Self-reported increase in willingness to explore personal grief. Improvement in sleep quality, self-confidence, increased access to trauma-related emotions and memories.
Van Gelderen et al. (2020b)	Examine experiences with 3MDR and underlying mechanisms of its effect.	Dutch soldiers with TR-PTSD	*N* = 10	Thematic analysis based on grounded theory	Themes: Engaging, stress regulation, support, encountering traumatic memories, allowing emotions, initiating associations, detachment from trauma. Increased openness, new learning, self-understanding, experience of reconciliation and reintegration.
Hamilton et al. (2021)	Investigate experiences with 3MDR and underlying mechanisms of its effect.	Canadian soldiers with TR-PTSD	*N* = 13	Thematic analysis	Themes: Importance of therapist’s support, benefits of the multimodal nature of 3MDR, experience of active participation, self-determination, increased access to emotions, and improved emotion regulation.
Tang et al. (2021)	Examine the effectiveness of 3MDR in improving emotion regulation and TR-PTSD.	Canadian soldiers with TR-PTSD	*N* = 9	Mixed methods design	Quantitative: Significant improvement in problematic emotion regulation, with an average reduction of 16 points after 3 months. Qualitative: Better emotional awareness, clarity, acceptance, and improved strategies.
Smith-MacDonald et al. (2023)	Explore the occurrence of moral injury in conjunction with TR-PTSD and perspectives on using 3MDR as a treatment for moral injury.	Canadian soldiers with TR-PTSD	*N* = 11	Mixed methods design	Quantitative: Significant relationship between TR-PTSD and moral injury.Qualitative: Descriptions of encountering the realities of war, grappling with conscience, and formation of moral meaning.
Hoekstra et al. (2023)	Explore the suitability of 3MDR for adolescents.	Dutch adolescent with TR-PTSD	*N* = 1	Case study	No-longer PTSD diagnosis post-intervention and at 18 months follow-up.

### 3.1 The effect of 3MDR treatment

Jetly et al. (2017) conducted the first study examining the effects of 3MDR on TR-PTSD. The participants consisted of male Canadian soldiers with TR-PTSD (*N* = 8). This pilot study was conducted without a control group to assess the need for larger randomized controlled trials. Three participants dropped out for various reasons, leaving a remaining sample of five participants who completed the standard 3MDR protocol. PTSD symptoms were measured using the PCL-5 questionnaire, and other PTSD-related outcome variables were assessed. Results were obtained by administering these questionnaires immediately before and after the completion of the 3MDR protocol. The study observed reductions in PTSD symptoms on the PCL-5 (an average reduction of six points). Regarding secondary outcome variables, most participants reported improvements in social functioning (OQ-45.2), thought suppression (WBSI), avoidance behavior (PABQ-5), and anxiety symptoms (GAD-7). These results were described as modest, and the study concluded that they provide a basis for conducting studies with larger samples and control groups.

Similarly, Bisson et al. (2020) conducted a randomized controlled trial to investigate the effect of 3MDR on TR-PTSD in British soldiers. The participants (*N* = 42) were evenly divided between the control and treatment groups (*N* = 21), and all participants were male. The treatment group underwent the standard 3MDR protocol. Outcome measures were obtained before, immediately after, and at 12 and 26 weeks post-treatment. The primary outcome variable (PTSD symptoms) was assessed using the CAPS-5 and PCL-5 questionnaires. All administrators of the questionnaires and tests were blind to the participants’ group allocation. The study observed a significant reduction in PTSD symptoms for the treatment group, with an average reduction of 17.7 points that persisted at the 26-week follow-up (Bisson et al., 2020, pp. 147–148). The treatment group showed an estimated symptom reduction of 37% on average, with a moderate to large effect size. The study had a dropout rate of 14% (Bisson et al., 2020, p. 148). The authors concluded that this suggests a promising future for 3MDR as a treatment option but cautioned against recommending the treatment in routine practice before the study is replicated (Bisson et al., 2020, p. 149).

To further investigate the effectiveness of 3MDR, van Gelderen et al. (2020a) conducted a similar randomized controlled trial, but with an active control group receiving non-trauma-focused psychotherapy. The study included the same outcome variables and treatment protocol. The participants were Dutch soldiers with TR-PTSD (*N* = 43), all but one male. Measurements were taken before, immediately after, and at 12 and 16 weeks post-treatment protocol. All administrators of the questionnaires and tests were blind to the participants’ group allocation. The dropout rate in the study was described as low (7%) for a study utilizing exposure-based methodology (van Gelderen et al., 2020a, p. 224). Significant reductions in PTSD symptoms, with a large effect size, were observed on the primary outcome variables (PCL-5 and CAPS-5). The reductions persisted and showed improvement up to 16 weeks. There were no significant results on the secondary outcome variables, except for reductions in anxiety symptoms (GAD-7). The study concluded that the results suggest that 3MDR is an effective treatment for TR-PTSD, however the study gave some, but not enough insight regarding the mechanisms through which 3MDR appears to work, necessitating further investigation (van Gelderen et al., 2020a, p. 225).

Jones et al. (2022a) conducted a similar study to replicate the results of Bisson et al. (2020) and van Gelderen et al. (2020a), but also further examined the specific aspects of 3MDR that contribute to its effectiveness. This study included additional secondary outcome variables related to PTSD and a longer follow-up period. Data were collected before 3MDR therapy, immediately after, and at 1, 3, and 6 months later. The sample (*N* = 11) consisted of Canadian soldiers with TR-PTSD, all but one male. Due to COVID-19 restrictions, control group data were unavailable (Jones et al., 2022a, p. 5). Nevertheless, participants who completed the 3MDR protocol showed statistically significant improvements in PTSD scores (CAPS-5 and PCL-5), moral injury (MISS-M-SF), depression (PHQ-9), anxiety (GAD-7), emotional regulation (DERS-18), and resilience (CD-RS-25). The improvements persisted up to 6 months after the 3MDR intervention. The study concluded that the significant results in secondary outcome variables provide some insight into underlying mechanisms but emphasized the need for further theory and research to address the current lack of understanding regarding the mechanisms underlying the apparent effectiveness of 3MDR (Jones et al., 2022a, pp. 9–10).

### 3.2 Individual experiences with 3MDR

Van Gelderen et al. (2018) conducted a case study with three Dutch soldiers with TR-PTSD, Nick, Luke, and Peter, with the aim of exploring 3MDR as a treatment method for TR-PTSD based on the participants’ individual experiences. Luke experienced a 25-point reduction in PTSD symptoms on the PCL-5, no longer meeting the criteria for a PTSD diagnosis (van Gelderen et al., 2018). This reduction in PCL-5 score continued to decrease by six points at the 6-month follow-up assessment. Nick reported benefiting from 3MDR, including experiencing an emotional breakthrough and recalling new aspects of his traumas. However, this was not reflected in his PCL-5 score, as it increased by eight points. It should be noted that Nick’s wife had passed away just before his final 3MDR session. Peter reported walking as a particularly important aspect of 3MDR and experienced a 30-point reduction on the PCL-5, no longer meeting the criteria for a PTSD diagnosis (van Gelderen et al., 2018). Overall, the participants’ feedback suggests that walking helped them overcome avoidance and maintain focus, while the use of multiple senses made it easier for them to engage in treatment, access traumatic memories, and process the associated emotions. They also emphasized the importance of addressing multiple traumatic memories simultaneously and engaging in post-session discussions with the therapist to facilitate better processing of the memories (van Gelderen et al., 2018).

In a similar case study by Nijdam and Vermetten (2018), no quantitative measurements were conducted, but the Dutch soldier, John reported improvements in PTSD symptoms. John had previously tried EMDR therapy and cognitive-behavioral therapy without experiencing improvement. Although John had been motivated for therapy, behavioral and cognitive avoidance hindered his progress with other exposure-based methods. However, this changed when John tried 3MDR, emphasizing that the feeling of actively “walking into the memories” was particularly important. He believed this allowed him to access the emotions related to his traumas. John also reported improved sleep quality, increased self-confidence, a welcomed experience of positive thoughts, and a strong willingness to confront his own grief.

No studies have investigated the effect of 3MDR therapy for non-military veterans with TR-PTSD until recently, where Hoekstra et al. (2023) conducted a case-study on a Dutch 14-year-old adolescent named Petra who was experiencing TR-PTSD in relation to a severe car-accident and mother’s drug abuse. Personally chosen music and images proved to be a suitable combination for Petra, fostering increased involvement in and commitment to the intervention and supported Petra to express and experience the emotions that were associated with the traumatic events she had been involved in (Hoekstra et al., 2023). PTSD-symptom severity was assessed using CAPS-5. Post-therapy, she no longer satisfied the criteria for PTSD, and this persisted at the 18-month follow-up assessment. Petra conveyed that PTSD-symptoms were persistently minimal, and that her traumatic events had transformed into organized memories. Reflecting on the 3MDR treatment, she described it as overwhelming and emotionally intense because unlike in her EMDR sessions, she could not avoid or pretend like she had done in EMDR sessions. Also, in contrast to the EMDR session, she did not worry about the impression she was making on the therapist during 3MDR (Hoekstra et al., 2023). This lack of concern reportedly facilitated a greater sense of openness, allowing her to let go of defenses.

### 3.3 Potential working mechanisms

Van Gelderen et al. (2020b) conducted a thematic analysis based on grounded theory to examine the perspectives of 10 participants on the processes and effects of 3MDR therapy. The participants were male Dutch soldiers with TR-PTSD. Regarding working mechanisms, the majority of participants described 3MDR as engaging and explicitly mentioned that it prevented the use of avoidance strategies, particularly due to the walking component (van Gelderen et al., 2020b). A prominent theme that emerged was “encounters with traumatic memories,” with most participants reporting a complete confrontation with their traumatic memories. This encounter was described as significantly more powerful than imaginal exposure, and was attributed to the self-selected images, music, being able to walk toward trauma-related images and the immersive experience that was created. Another prominent theme was “permission of emotions,” several participants reported the emergence of entirely new emotions, and some highlighted the importance of therapist questioning in this process. Under the theme of “association,” several participants mentioned the significance of trauma-related images in initiating new associations. Many participants also described a sense of “detachment from trauma” and emphasized how the dual-attention task and music contributed to reconciliation. The support provided by the therapist standing by their side was also reported to facilitate all therapy processes.

The experienced effects formed the following themes: increased openness, new learning, self-understanding, experience of closure, and reintegration (van Gelderen et al., 2020b, pp. 8–9). The theme *increased openness* refers to participants’ improved willingness to discuss their traumatic experiences with others. Several participants reported experiencing a newfound understanding that they can feel safe. Participants also reported changes in thoughts and feelings based on this new learning. This relates to the theme of *self-understanding*, where participants gained better insight into the people or situations they perceived as triggers, leading to improved symptom control. Participants also described a sense of *closure*, experiencing their memories as more organized and meaningful than before treatment. Most participants also reported that 3MDR helped them reintegrate to society. As an example, many of the participants reported being able to engage in normal activities again, like playing football or going to cafes.

Hamilton et al. (2021) conducted a qualitative thematic analysis with 13 Canadian soldiers or veterans with TR-PTSD, all but one men. Three themes emerged from the data: (1) participants’ experiences with 3MDR; (2) perceived outcomes of 3MDR; and (3) factors contributing to successful 3MDR treatment (Hamilton et al., 2021, pp. 1–2). Participants described 3MDR as providing an immersive environment that fostered active participation and increased autonomy. This enabled them to access emotions that had been inaccessible and provided insight into previously unexplored aspects of their traumatic experiences. Several participants reported that this newfound awareness facilitated better emotional regulation.

Themes that emerged as contributing factors to successful treatment included the therapist’s role as a coach and “fireteam partner,” supporting participants’ sense of safety and control over their therapy (Hamilton et al., 2021). The multimodal nature of 3MDR was also described as crucial in helping participants engage and attribute new meaning to traumatic memories. Several participants also expressed the belief that it was important to have tried other treatments before 3MDR. This is because 3MDR can be highly overwhelming if individuals have not previously experienced less realistic forms of exposure (Hamilton et al., 2021). Moreover, several participants described how active-duty military personnel might be reluctant to engage in such treatment, as opening these traumatic memories could potentially impact their effectiveness as soldiers. Additionally, it was noted that the act of having “tried everything” seemed reconciliatory, as several participants reported that this was the last treatment they were willing to attempt.

Tang et al. (2021) conducted a mixed methods study to investigate whether 3MDR improves emotion regulation and, consequently, TR-PTSD. The participants consisted of nine Canadian male soldiers with TR-PTSD. A quantitative examination of how 3MDR affects emotion regulation (DERS-18 score) was conducted immediately after, 1 week after, and 3 months after the treatment. Furthermore, a qualitative descriptive analysis of the data was performed. In the quantitative analysis, a significant improvement in symptoms of problematic emotion regulation (DERS-18) was observed, with participants showing a steady decline in symptoms up to 3 months post-intervention.

The qualitative data collected were categorized according to their relevance to the DERS-18 subcategories. The data suggested that participants expressed noticeable improvement in all DERS-18 subcategories. Participants reported the most significant changes in “awareness,” “clarity,” “non-acceptance,” and “strategies” (Tang et al., 2021, pp. 7–8). A recurring description was that 3MDR allowed access to emotions that were previously inaccessible, and the therapist was described as crucial in verbalizing these emotions. Participants expressed that gaining awareness of their feelings, thoughts, and experiences was essential for the improvement in emotion regulation, making it easier to develop effective emotion regulation strategies. Acceptance appeared to be a strategy that many participants reported as particularly important following 3MDR.

Smith-MacDonald et al. (2023) conducted a mixed methods study uniquely aimed at investigating (1) whether moral injury occurs concurrently with TR-PTSD in military personnel and (2) the perspectives of military personnel with TR-PTSD on 3MDR as a treatment for moral injury and PTSD (Smith-MacDonald et al., 2023, pp. 2–3). The study employed quantitative and qualitative data collection methods to explore these aspects. The participants were Canadian soldiers with TR-PTSD (*N* = 17). However, four participants were unable to complete the treatment due to COVID-19 restrictions (Smith-MacDonald et al., 2023, p. 4). Two other participants did not complete the treatment for other reasons, leaving 11 remaining participants for data analysis. In the quantitative analysis, reductions in PTSD symptoms (CAPS-5 and PCL-5) were examined in relation to symptoms of moral injury (MISS-M-SF). Significant reductions in PTSD symptoms were observed. Additionally, PTSD-related concepts such as depression symptoms (PHQ-8), anxiety symptoms (GAD-7), psychological functioning (OQ-45.2), psychological resilience (C-DRS), alcohol problems (AUDIT), and emotion regulation (DERS-18) were measured. A significant relationship between the decrease in moral injury and PTSD symptoms (CAPS-5 and PCL-5) was observed (Smith-MacDonald et al., 2023). Furthermore, there was a significant association between moral injury and all PTSD-related concepts, except alcohol problems.

In the qualitative analysis, a thematic analysis of data collected through semi-structured interviews was conducted. Three overarching themes were identified: (1) Realities of war, (2) Wrestling scruples, and (3) Creation of moral sensemaking (Smith-MacDonald et al., 2023, pp. 9–11). Regarding the theme “realities of war,” several participants described a narrative in which they buried and suppressed emotions that conflicted with their values and experiences. Participants expressed that being in war was not as they had expected, having to blindly follow orders and prioritize the mission over what they perceived as morally right. Deviating from their role could put their comrades, troops, friends, and civilians at risk. As a result, many participants felt helpless, angry, and confused. Several participants reported creating a “black box” to store emotions and reactions that could affect their performance during missions.

Central to the theme “wrestling scruples” was participants’ experience of unresolved dissonance between what had happened to them and what should have happened to them. They reported feelings of shame and guilt resulting from being exposed to conflicting emotions and experiences over an extended period (Smith-MacDonald et al., 2023).

The theme “moral sensemaking” relates to participants’ experiences of 3MDR therapy assisting in sorting out and giving meaning to their experiences, actions, and choices (Smith-MacDonald et al., 2023). Participants reported being able to engage with the content of the “black box” mentioned earlier. They described transitioning from a black-and-white perspective on their experiences and emotions to a more pragmatic perspective after 3MDR. Participants expressed a form of acceptance toward what had happened to them and the emotions they had regarding it.

The study concluded that although the results are preliminary, they observed how moral injury often co-occurs with TR-PTSD in military veterans. Additionally, 3MDR appears to be an effective treatment for moral injury and TR-PTSD (Smith-MacDonald et al., 2023, p. 14).

## 4 Discussion

The studies included consistently demonstrate significant effects of 3MDR on TR-PTSD. This finding is surprising considering the severity of PTSD that was examined in the studies. In the study by van Gelderen et al. (2020a), participants had, on average, undergone four forms of psychological treatment and three pharmacological treatments without experiencing clinical improvement. The participants in the study by Bisson et al. (2020) exhibited a similar level of severity, with all participants having undergone a minimum of two trauma-focused therapy approaches without experiencing clinical improvement. Furthermore, all included studies had similar samples, but Bisson et al. (2020) and van Gelderen et al. (2020a) are highlighted due to their larger sample sizes and inclusion of control groups. The studies with a randomized controlled design provide the most robust evidence regarding the effectiveness of 3MDR. The case studies and the quantitative studies that did not utilize a control group also suggest a positive effect of 3MDR on TR-PTSD and further highlights the specific aspects of 3MDR that might contribute to efficacy.

### 4.1 Working mechanisms

In the qualitative studies presented, it emerges that 3MDR facilitated access to memories and emotions that had not yet been available. In Hoekstra et al. (2023), for example, Petra reported that her self-selected music had a very special role in enabling access to previously inaccessible emotions. In van Gelderen et al. (2020b), it was also specifically mentioned that the combination of self-selected music and images was crucial for this access. As presented in the introduction, a challenge with exposure therapy has been to find the right type of stimuli to activate the client’s traumatic memories. The fact that participants themselves are involved in the process of choosing stimuli (images and music) increases the likelihood that the stimuli used can be related to the correct memories. Being in a screen room where one experiences “walking into memories” may increase immersion. Additionally, this process is repeated with several different images, which may contribute to the activation of multiple related memory networks. This can increase the likelihood of adequately activating the traumatic memory, allowing for the organization of the overall memory and the incorporation of new learning.

#### 4.1.1 The therapist

In nearly all the qualitative studies, the therapist was reported as being important for facilitating all aspects of successful treatment. As an example, the most frequently mentioned theme for efficacy in the study of van Gelderen et al. (2020b) was the support of the therapist. In the study of Hamilton et al. (2021), the therapeutic alliance was highlighted as particularly important as the experience of having someone beside you while walking “through trauma” was deemed as providing a safe atmosphere where therapists could challenge avoidance behaviors. Some participants went as far as saying the therapy would not have worked if it was not for the particular therapist they were assigned, suggesting the therapeutic alliance was very important (Hamilton et al., 2021). Just as in other forms of trauma-therapy, it seems that the therapist plays an important role for successful treatment. It could be argued that 3MDR is a complementary protocol for enhancing currently accepted trauma-therapies rather than being an “alternative” to current trauma therapies. The therapist and the techniques used are seemingly important and 3MDR therapy might not show efficacy if not used in conjunction with experienced therapists, trained in trauma-therapy. The immersive nature of the 3MDR protocol enhances aspects of already established trauma-therapies by addressing problems with gaining access to trauma and decreasing avoidance. 3MDR could be used for creating a context that facilitates doing the actual work with the traumatic experiences/memories if patients are having trouble working through trauma. When a breakthrough has been facilitated by 3MDR, going back to other forms of non-VR-assisted trauma therapies for further processing of what has come up during 3MDR therapy might be ideal.

#### 4.1.2 The organizing nature of 3MDR

As previously noted, several participants in the qualitative studies reported accessing emotions and memories previously inaccessible to them. This suggests presence of unprocessed and unintegrated memories and emotions, aligning with EPT’s concept of isolated fear structures. Smith-MacDonald et al. (2023) highlighted participants’ accounts of accessing the “black box” contents and subsequently integrating them in a manner conducive to a more organized understanding of their experiences. Similar observations of organizational and interpretive experiences were noted in van Gelderen et al.’s (2020b) study. It appears plausible that 3MDR not only facilitates access to trauma but also incorporates functions for organizing and integrating trauma content. It is conceivable that articulating participants’ emotional responses and recollections on the 3MDR screen could serve as an organizational feature. Moreover, the therapist’s presence may further provide tools for organization and support in verbalizing experiences.

#### 4.1.3 Movement

The movement provided by treadmill-walking in the 3MDR protocol may have several benefits for integrating new experiences/learning, providing access to trauma content and reducing avoidance. Several studies suggest that brain-derived neurotrophic factor (BDNF) levels are crucial for synaptic plasticity in the amygdala, and thus, BDNF levels may play a critical role in fear learning and extinction (Chhatwal et al., 2006; Bredy et al., 2007). For instance, in a study conducted by Chhatwal et al. (2006) on mice, reduced BDNF levels were found to impede the extinction of fear responses. This aligns with multiple studies proposing that BDNF plays a significant role in learning and memory (Yamada et al., 2002; Bekinschtein et al., 2014; Nilsson et al., 2020). This suggests that increasing BDNF levels (through physical activity) during therapy may contribute to enhanced consolidation of new learning or extinction. Additionally, it has been observed that walking can lead to increases in BDNF levels, suggesting that the 45–60 min of treadmill walking in 3MDR may contribute to similar increases (Merom et al., 2008).

Some research also suggests that walking may increase divergent thinking, therefore increasing associations (Oppezzo and Schwartz, 2014). For example, a study by Oppezzo and Schwartz (2014) found that participants could generate twice as many uses for an object while walking on a treadmill compared to when they were sitting still. This suggests that treadmill walking, as in 3MDR, may facilitate increased trauma associations and therefore greater access to trauma.

In the study of van Gelderen et al. (2020b), participants described how walking encouraged them to confront their traumatic memories. This can be related to the research literature on embodied cognition, which can be defined as “how bodily states affect mental states” (Wilson and Golonka, 2013, p. 1). In this regard, it has been observed that physically approaching a fear-inducing stimulus results in a more positive evaluation of the object (Woud et al., 2008; Wolitzky and Telch, 2009). In 3MDR, participants may experience the same effect, which could further reduce the likelihood of participants using avoidance strategies, aligning with what participants reported in the study of van Gelderen et al. (2020b).

### 4.2 Moral injury

Moral injury describes the psychological harm that can occur when individuals commit, witness, or fail to prevent an action that violates their core values (Litz et al., 2009; Smith-MacDonald et al., 2023, p. 2). As suggested by Smith-MacDonald et al. (2023), moral injury is seemingly more prevalent in military veterans, which in turn might explain the higher prevalence of TR-PTSD in military veterans. Potentially morally injurious events are particularly relevant for individuals who have been in war zones (military), as the likelihood of being part of such events is higher (Battles et al., 2018). Military veterans have been observed to respond less favorably to existing evidence-based PTSD treatments compared to civilians, providing support for an idea that there are aspects of their traumatic experiences that are of a different nature (Steenkamp et al., 2015). It is therefore possible that TR-PTSD and moral injury are closely linked. In the study of Smith-MacDonald et al. (2023), measures of moral injury symptoms were significantly reduced by 3MDR therapy and the authors therefore presumed that some of the efficacy of 3MDR could be explained by its ability to treat individuals with moral injury. They believe that the traumatic content in individuals with moral injury is harder to access and that 3MDR facilitates reduced avoidance and increased immersion, which subsequently leads to reduced moral injury symptoms. The relationship between TR-PTSD and moral injury, however, remains unclear, and although the concepts have shown to correlate, study of Smith-MacDonald et al. (2023) had few participants and lacked a control group. Additionally, it is unclear whether moral injury can be considered as a part of PTSD or as a separate concept (Koenig et al., 2020). As of now moral injury is not an official diagnosis. Much of what is currently labeled as PTSD may therefore be moral injury. It is therefore not possible to determine with certainty whether 3MDR has a specific mechanism of action mediated by reduction of moral injury symptomatology, and the results should therefore be interpreted with caution.

### 4.3 Drop-out rates

As mentioned in the introduction, drop-out has been a problem in therapy approaches that utilize exposure-based methodology. According to a meta-analysis by Kitchiner et al. (2019), drop-out rates range from 16 to 31% for EMDR and 5 to 44% for VRET. The results for 3MDR appear promising in this regard, as van Gelderen et al. (2020a) reported a 7% drop-out in their study, and Bisson et al. (2020) reported a 14% drop-out rate. Based on this, 3MDR demonstrates a strong position with drop-out rates between 7 and 14%. However, when including smaller, non-controlled quantitative studies of 3MDR, slightly higher drop-out rates are observed. Smith-MacDonald et al. (2023) reported a 35% drop-out rate, and Tang et al. (2021) reported a 33% drop-out rate (for an overview, see Table 2). It is important to note that a significant portion of the drop-out was due to COVID-19 restrictions. Much of the 3MDR research was conducted during the pandemic, resulting in delays. Nonetheless, when comparing drop-out rates with VRET and EMDR, 3MDR demonstrates lower drop-out rates. The drop-out rates reported for EMDR and VRET in Kitchiner et al.’s meta-analysis were based on studies examining non-chronic PTSD. In the 3MDR studies, the samples consist of individuals with chronic PTSD (TR-PTSD), suggesting these individuals are more likely to drop out. The lower drop-out rates for 3MDR therefore suggests that 3MDR might reduce avoidance behavior in subjects. As mentioned in the “working mechanisms” section there may be several components of the 3MDR protocol that reduce avoidance.

**Table 2:** Drop-out rates for quantitative studies.

Authors	Dropout rates
Van Gelderen et al. (2020a)	7%
Bisson et al. (2020)	14%
Tang et al. (2021)	33%
Jetly et al. (2017)	37,5%
Smith-MacDonald et al. (2023)	35%
Average:	25,5%

### 4.4 Comparison with other treatments

There is a lack of comparative basis for assessing the effectiveness of 3MDR against other forms of psychotherapy, as few evidence-based therapies have been investigated for treating TR-PTSD (Koek et al., 2016). However, promising results have been observed for methylenedioxymethamphetamine (MDMA)-assisted psychotherapy for TR-PTSD, with large effect sizes reported in some studies (Mithoefer et al., 2018; Ot’alora G et al., 2018). Methods such as deep brain stimulation (Koek et al., 2019; Hamani et al., 2020), ketamine assisted trauma therapy (Feder et al., 2014, 2021) and optogenetics also show promise in treating TR-PTSD in the future, but as for now, these therapies have not been systematically studied for TR-PTSD (Eyraud et al., 2023; Xi et al., 2023).

All the aforementioned treatment alternatives for TR-PTSD are pharmacological or invasive in nature. Given that current studies on 3MDR demonstrate moderate to large effect sizes and exclusively significant results, 3MDR may prove to be a useful non-invasive and non-pharmacological addition to the clinicians’ toolkit. In light of the literature on ketamine, optogenetics, and deep brain stimulation, 3MDR appears to have the strongest evidence base thus far, as only case studies have been conducted on deep brain stimulation, optogenetics relies on animal experiments, and ketamine treatment has shown only short-term improvement. However, it can be argued that the evidence base for MDMA-assisted psychotherapy is comparable to 3MDR.

### 4.5 Methodological limitations

The studies conducted on 3MDR are compelling, but these findings should also be interpreted with caution due to several methodological limitations in some of the studies.

#### 4.5.1 Generalizability of the findings

Several studies lack control groups and are constrained by being conducted under COVID-19 restrictions. Only Bisson et al. (2020) and van Gelderen et al. (2020a) have conducted randomized controlled trials (RCTs) with a larger number of participants. While these studies do not have few participants, they are also not large-scale compared to similar RCT studies on trauma-focused treatment methods (Kitchiner et al., 2019). The fact that the studies yield similar results is promising. However, there is a high homogeneity in the samples of the 3MDR studies, as all of them examine the same type of sample (soldiers with TR-PTSD). Additionally, few studies include female participants, and when they are included, they are only represented by a small part of the sample, such as in van Gelderen et al. (2020a) study where only one out of 42 participants were female. Studies with more representative samples are necessary before generalizing the findings to other populations.

The samples used in the studies also exclusively consist of individuals with severe forms of PTSD (TR-PTSD). It would be interesting to see how a representative civilian population with varying levels of PTSD severity responds to the treatment. Nevertheless, it appears that male soldiers with TR-PTSD benefit from 3MDR. However, generalizability is likely to be limited to soldiers who are open to PTSD treatment and not to those who have given up on treatment.

#### 4.5.2 Confounding variables

In the studies that utilized quantitative measurements, the assessors of the measurement scales were blinded to whether the participants had undergone 3MDR therapy or not. However, blinding the participants to the treatment condition was not possible. The experience of being assigned to this innovative treatment may have influenced the participants’ expectations, which could have resulted in reduced PTSD symptoms. However, all included studies conducted follow-up measurements several weeks and months after treatment, which still showed reductions in PTSD symptoms. This continued symptom reduction argues against a placebo effect being the sole factor contributing to the effectiveness of 3MDR, although it could potentially be a contributing factor.

In both qualitative and quantitative studies, demand characteristics may have influenced the results. Demand characteristics refer to participants being aware of what the researchers are investigating and being inclined to responses that align with the intended purpose (McCambridge et al., 2012). 3MDR has distinct elements that participants may pick up on as important to the researchers. Therefore, the qualitative interviews conducted may be vulnerable to participants’ reporting therapeutic processes and effects that align with what they believe the researchers wanted to hear. As suggested in Hamilton et al. (2021) study, several participants reported that 3MDR was the last treatment they were willing to try, and therefore perceived this as reconciliatory. This sense of reconciliation may have contributed to the observed effects of 3MDR.

In all the included studies, patients received other treatment alongside 3MDR therapy. For instance, in the study by van Gelderen et al. (2020a), participants received an average of 4.56 therapy sessions within 10 weeks after 3MDR treatment (van Gelderen et al., 2020a, p. 223). Therefore, it cannot be definitively determined that the observed changes in 3MDR can be solely attributed to 3MDR. However, it seems unlikely that the additional follow-up therapies significantly contributed to the main effects, considering that the samples consist of individuals who have previously not responded to treatment.

### 4.6 Implications for further research

While 3MDR shows promising results for soldiers with treatment-resistant PTSD, caution should be exercised in assuming that this implies 3MDR should be recommended as a treatment method. Further research is necessary to assess whether 3MDR can be applied to populations other than those investigated thus far. Two critical questions are pivotal for the future of 3MDR: (1) whether populations without treatment resistance can benefit from 3MDR, and (2) whether the costs of 3MDR justify the observed effects.

It is not self-evident that 3MDR is effective for populations without treatment resistance, even though the therapy demonstrates positive effects on treatment-resistant PTSD. For example, participants in the study of Hamilton et al. (2021) reported the importance of attempting other treatments before 3MDR, as 3MDR entails overwhelming exposure. While there is a theoretical goal of activating trauma memories to treat PTSD, it would be inappropriate to activate individuals who are not ready to address these memories. This could potentially worsen their condition if the memories are not treated and organized adequately while they are active. Individuals with treatment-resistant PTSD have already undergone numerous treatments and are likely familiar with much of their trauma content. Hence, there may be a lower likelihood of 3MDR opening “wounds” that are too difficult to close in these individuals. However, in individuals without TR-PTSD there might be a risk of overwhelming the client in a way that leads to worsening of their condition. Furthermore, the studies conducted thus far have almost exclusively focused on military personnel. It is conceivable that their traumas differ from those found in civilian populations with PTSD. Civilian populations may react differently to 3MDR, necessitating further research to explore this aspect.

Apart from military veterans, other target groups for the therapy could be survivors of other severe traumas leading to TR-PTSD, such as terrorist attacks and car accidents. Hoekstra et al. (2023) based on the results of their case study suggest that 3MDR may be well suited for young patients with TR-PTSD, as they are used to extensive exposure to the digital world and self-selected music and images for repeated exposure may feel natural to them. It remains to further investigation to establish to which extent 3MDR could help patients with incomplete memories (amnesia) for the traumatic events they were exposed to; van Gelderen et al.’s (2018) case study indicated that 3MDR enabled recalling new aspects of traumas.

In the context of exposure therapy, there is a concern regarding secondary traumatization (STS), which refers to the distress or PTSD symptomatology that therapists may experience due to exposure to the traumatic experiences of their clients (Figley, 2015). In VRET, clients are immersed in a VR headset, limiting the therapist’s direct exposure to graphic imagery. In contrast, with 3MDR, therapists are actively involved in the virtual experience alongside the client. This raises the hypothesis that 3MDR therapists may be more susceptible to secondary traumatization. However, a recent qualitative study by Jones et al. (2022b) explored the impact of STS on 3MDR operators and therapists. Despite therapists and operators (*N* = 13) reporting some impact, they perceived little to no difference compared to other trauma therapies. While this study provides valuable insights, its small scale suggests the need for further research to thoroughly examine how therapists and operators are affected by this mode of delivering trauma therapy.

Military veterans with TR-PTSD may have sustained injuries leading to various handicaps as a consequence of deployment to warzones. Further research should therefore investigate the possibility of making 3MDR more accessible for handicapped individuals who may face challenges using a treadmill. While the treadmill component is deemed important, it may not be indispensable for successful 3MDR treatment. A considerable number of participants in qualitative 3MDR studies have emphasized the experience of moving toward trauma-related content as a crucial aspect of treatment-efficacy. Thus, it would be valuable for forthcoming studies to explore the feasibility of implementing the 3MDR protocol using a treadmill that accommodates individuals who use wheelchairs. This could involve the utilization of a wheelchair on a specially adapted treadmill, combined with a different type of safety harness.

In addition to assessing clinical effectiveness, studies on cost-effectiveness are necessary. 3MDR is a costly intervention; the equipment is expensive, and it requires substantial resources in terms of therapist’s time and equipment. A 3MDR session lasts for 90 min, requiring an operator to control the 3MDR platform and a therapist (van Gelderen et al., 2020a). A screen room with a treadmill and appropriate software is also necessary to conduct 3MDR therapy. Consequently, 3MDR may struggle to become a treatment method offered to the majority of PTSD cases. A more plausible scenario could be that this treatment is offered as a last resort for individuals who have not benefited from previous treatments. This may explain why 3MDR studies primarily focus on individuals with treatment-resistant PTSD. Nevertheless, a health economic evaluation should be conducted to enable informed treatment decisions and recommendations.

A plausible strategy to enhance the cost-effectiveness of 3MDR therapy could involve leveraging artificial intelligence (AI) technologies. Specifically, speech-to-text technologies could automate the process of projecting associations verbalized by the patient, eliminating the need for a session operator to project patient associations onto the screen. Additionally, equipping therapists with tablets could empower them to independently administer trauma-related stimuli. If proven feasible in later 3MDR versions, these AI-driven enhancements and changes to the setup could significantly improve the therapy’s cost-effectiveness.

## 5 Conclusion

Psychotherapy utilizing exposure-based methodologies have proven effective for PTSD. However, a significant proportion of individuals have dropped out of multiple trauma-focused treatment methods and are therefore considered to have treatment-resistant PTSD. Research on alternative treatment methods for this group has been limited. This literature review explored 3MDR therapy as an alternative for individuals with treatment-resistant PTSD, and preliminary studies indicate a positive effect of 3MDR. The mechanisms underlying this effect are not yet fully understood, but qualitative studies and relevant theory suggest potential mechanisms of action. The use of images and music in combination appears to provide access to previously inaccessible memories and emotions. 3MDR also appears to have an organizing effect on trauma memories, with the therapist playing a key role. The studies also suggest that movement toward trauma memories may contribute to the consolidation of new learning, increases in trauma associations and reduction in avoidance. Support has also been found for 3MDR therapy reducing symptoms of moral injury and maladaptive emotion regulation, but these findings are limited by methodological challenges and require further investigation. Research on 3MDR is still in its early stages, and the studies presented have relatively small and homogeneous samples. More controlled studies with larger and more diverse samples are necessary. Furthermore, future studies should evaluate cost-effectiveness of 3MDR and examine the effect of 3MDR in different civilian populations with various degrees of PTSD severity.

## Author contributions

ÅG: Writing – original draft, Investigation. PF: Writing – review & editing, Supervision, Methodology.

## Footnotes

^1^ https://oria.no/
